# The differences between patients with nonalcoholic fatty liver disease (NAFLD) and those without NAFLD, as well as predictors of functional coronary artery ischemia in patients with NAFLD

**DOI:** 10.1002/clc.24205

**Published:** 2023-12-18

**Authors:** Wen‐Jing Li, Hong‐Wei Xu

**Affiliations:** ^1^ Department of Medical Imaging Fifth Affiliated Hospital of Zhengzhou University Zhengzhou China

**Keywords:** CACS, CAD‐RADS, CCTA, CT‐FFR, NAFLD

## Abstract

**Background:**

Nonalcoholic fatty liver disease (NAFLD) is a chronic liver disease associated with metabolic syndrome. It is the most common cause of cryptogenic cirrhosis. The disease is also involved in the occurrence and development of type 2 diabetes and atherosclerosis and can directly affect the outcome of patients with coronary heart disease. Therefore, the focus of treatment of nonalcoholic fatty liver disease has also begun to focus on the treatment of risk factors for atherosclerotic heart disease. In this study, we investigated the difference between patients with coronary artery stenosis combined with NAFLD and those without NAFLD and evaluated the predictive factors and value of functional coronary artery ischemia in patients with NAFLD.

**Hypothesis:**

Many clinical factors (such as age, BMI, hyperglycemia) and imaging parameters (such as CACS grade) in the NAFLD group were different from those in the non‐NAFLD group. The predictive model combined with multiple influencing factors has a good value in predicting coronary artery ischemia in patients with NAFLD.

**Methods:**

We collected the clinical and imaging data of patients who underwent coronary computed tomography angiography and coronary artery calcification score (CACS) scans between January and June 2023. A total of 392 patients were included and divided into the NAFLD group and the non‐NAFLD group. Based on CT fractional flow reserve (CT‐FFR), patients with NAFLD were divided into CT‐FFR ≤ 0.08 group and CT‐FFR > 0.08 group.

**Results:**

Significant differences were observed between the non‐NAFLD and NAFLD groups in terms of age, body mass index, hyperglycemia, hyperlipidemia, triglyceride, high‐density lipoprotein, coronary artery disease‐reporting and data system (CAD‐RADS) classification, CACS classification, number of diseased coronary arteries, and CT‐FFR ≤ 0.80 ratio (*p* < .05). The CAD‐RADS and CACS classifications can independently predict functional coronary artery ischemia in NAFLD patients. The combined use of CAD‐RADS and CACS classifications resulted in an area under the curve of 0.819 (95% confidence interval: 0.761–0.876) for predicting coronary artery ischemia in NAFLD patients, which was higher than the individual classification methods (CAD‐RADS: 0.762, CACS: 0.742) (*p* = .000).

**Conclusions:**

There are differences between patients with coronary artery stenosis and NAFLD and those without NAFLD. The CAD‐RADS classification and CACS classification can economically and efficiently predict functional coronary artery ischemia in patients with NAFLD, which has crucial value in clinical diagnosis and treatment.

## Graphical abstract

Statistically significant difference factors between patients with nonalcoholic fatty liver disease (NAFLD) and those without NAFLD. Body mass index, the proportion of hyperglycemia and hyperlipidemia, UA, and TG of NAFLD patients were higher than those of the NO NAFLD group, while HDLC and Age were lower than that of NO NAFLD patients.

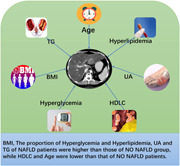

**Abbreviations**
AUC: areas under curve
BMI: body mass index
CACS: coronary artery calcification score
CAD‐RADS: coronary artery disease‐reporting and data system
CCTA: coronary computed tomography angiography
CPR: cardiopulmonary resuscitation
CT‐FFR: CT fractional flow reserve
FFR: fractional flow reserve
NAFLD: nonalcoholic fatty liver disease
ROC: receiver operating characteristic
VR: volume rendering

## 1 INTRODUCTION

Nonalcoholic fatty liver disease (NAFLD) is a chronic liver disease associated with metabolic syndrome and is the primary cause of chronic liver disease worldwide.^1^ This disease not only causes decompensated cirrhosis, liver transplant rejection, and hepatocellular carcinoma, but it also contributes to the occurrence and development of type 2 diabetes mellitus and atherosclerosis. NAFLD is associated with increased liver‐related incidence or mortality rates. Cardiovascular disease is the primary cause of death in patients with NAFLD.^2^ It is most closely associated with cardiovascular disease.^3^ Several stages of the occurrence and development of cardiovascular disease may be affected by inflammatory reactions and changes in lipid hormone levels in NAFLD, which have a direct impact on the prognosis of coronary heart disease.^4^, ^5^ Numerous studies have demonstrated that traditional risk factors for coronary heart disease, such as hypertension, dyslipidemia, obesity, and smoking,^6^, ^7^ are also risk factors for NAFLD. Other studies^8^ have shown that patients with NAFLD are more likely to develop coronary microvascular dysfunction than patients without NAFLD.

In 2016, the Society of Cardiovascular Computed Tomography released the coronary artery disease‐reporting and data system (CAD‐RADS) 1.0, which is widely recognized and utilized throughout the world.^9^ CAD‐RADS classifies patients based on coronary computed tomography angiography (CCTA) results, which represent the most severe level of coronary artery disease. Prior research^10^, ^11^ has demonstrated the high diagnostic accuracy and prognostic value of CAD‐RADS. In recent years, CT fractional flow reserve (CT‐FFR) has emerged as a focal point of clinical research and application. It can reliably identify coronary artery ischemic lesions with a single examination, assisting physicians in determining the vessels responsible for myocardial ischemia.^12^, ^13^, ^14^ Numerous studies have confirmed that CT‐FFR and invasive FFR have high consistency and accuracy in diagnosing specific ischemic lesions. coronary artery calcification score (CACS) is a noninvasive CT technique that detects and reflects the burden of coronary calcified plaque, and it is a favorable tool for the evaluation of coronary heart disease. Several investigations^15^ have indicated that liver diseases due to NAFLD are significantly associated with coronary artery calcification. In this study, we analyzed the difference between patients with coronary artery stenosis combined with NAFLD and those without NAFLD. We explored the predictive factors of functional coronary artery ischemia in patients with NAFLD based on CT‐FFR, providing a theoretical basis for early clinical diagnosis and risk stratification of coronary artery ischemia in patients with NAFLD.

## 2 DATA AND METHODS

### 2.1 Participants and data

A total of 921 hospitalized patients who underwent CCTA examinations and CACS scans at the Fifth Affiliated Hospital of Zhengzhou University between January 2023 and June 2023 were screened retrospectively. Exclusion criteria: (1) history of cardiac surgery, including percutaneous coronary intervention, coronary artery bypass grafting, cardiac pacemaker implantation, and valve replacement; (2) history of liver disease other than NAFLD, such as live cancer and cirrhosis; and (3) poor image quality of CCTA. Ultimately, 392 patients, including 209 patients combined with NAFLD and 183 patients without NAFLD, were enrolled to collect clinical and imaging data. NAFLD is defined as nonenhanced CT liver attenuation (liver–spleen CT values < 1 HU) or liver CT value/spleen CT value ≤ 1 HU, without clinical evidence of liver disease, cirrhosis, or alcohol abuse.^16^

### 2.2 CCTA and CACS scanning protocols

All patients underwent CCTA and CACS using a 256‐slice spiral CT scanner (Philips, Brilliance iCT). Patients received breath‐hold training before scanning to reduce motion artifacts, and those with high heart rates were given metoprolol to lower their heart rate below 65 bpm. To ensure adequate coronary artery dilatation, patients were instructed to take nitroglycerin sublingually within 2–5 minutes before scanning. CACS scanning was performed before a nonenhanced CT scan. The contrast agent iohexol (60–80 mL) was injected through the elbow vein at a flow rate of 4–5 mL/s using a double‐cylinder high‐pressure injector. Using the automatic contrast agent tracking threshold triggering technique, the trigger site was located between the ascending aorta and tracheal bifurcation. When the trigger threshold reached 150 HU, the scanning was delayed for 4.2 seconds. Subsequently, the CCTA images were reconstructed using iterative reconstruction technology, and the phase with the best image quality was selected for the postprocessing analysis of the CCTA. The CACS was automatically calculated by Agatston Calcium Score Software (Heart Beat‐CS) and divided into three categories: CACS = 0, 1–99, and ≥100.

### 2.3 CCTA characteristic analysis

**Figure 1:**
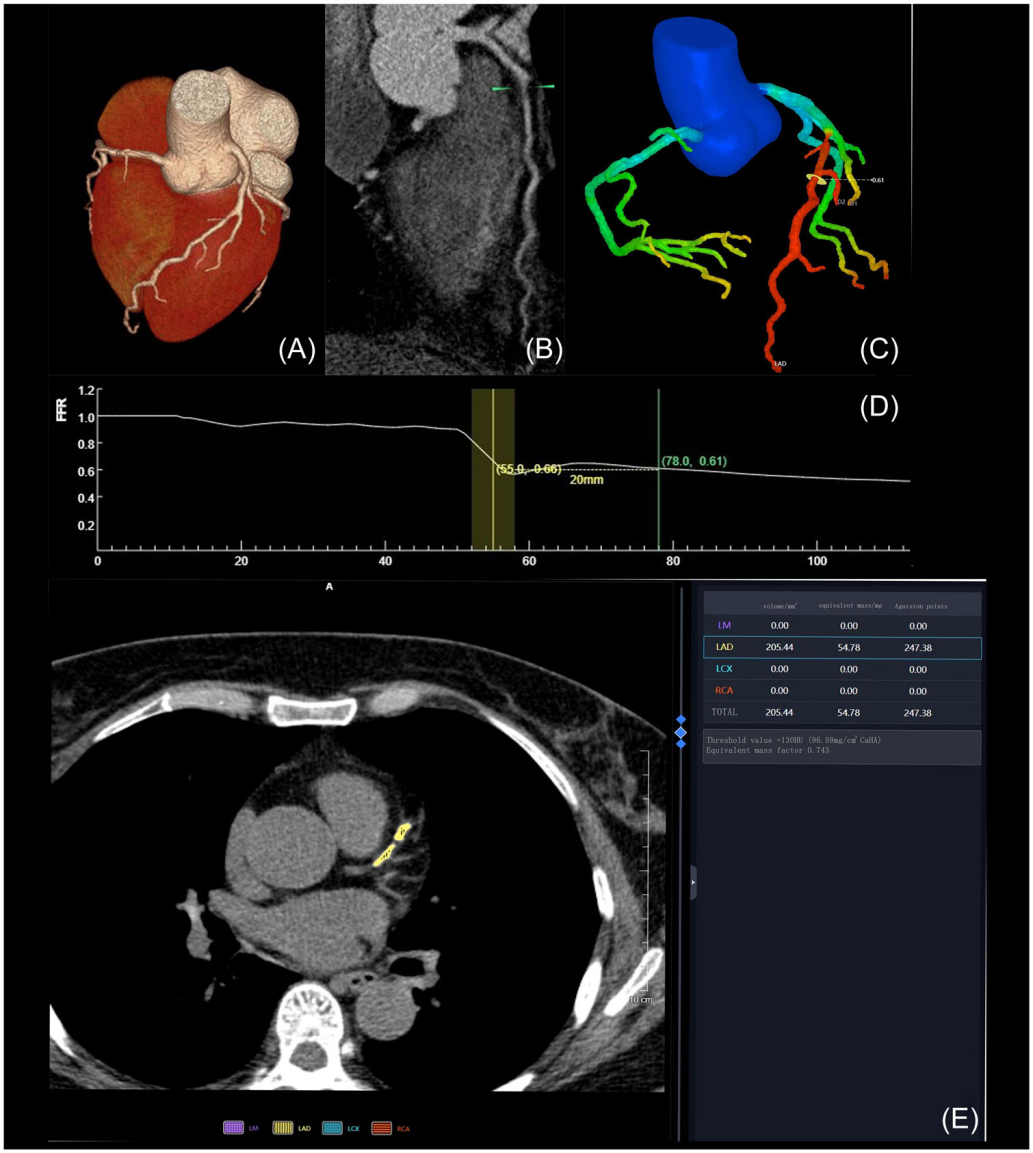
(A) Cardiac volume rendering image (VR); (B) computed tomography angiography curved planar reformation image; (C) VR coronary tree color map; (D) CT fractional flow reserve value curve; (E) coronary artery calcification score calculation interface.

The optimal image was transferred to the artificial intelligence software (United Imaging Intelligence, uAI Discovery‐Coronary CTA) to obtain the coronary CT‐FFR value. A total of 20 mm distal to the lesion, the CT‐FFR value was determined. CT‐FFR ≤ 0.8 indicates coronary artery ischemia, whereas CT‐FFR > 0.8 indicates absence of ischemia (Figure 1A–E). The CAD‐RADS classification was based on the results of CCTA,^9^ and its reference criteria were as shown in Table 1. In this study, CAD‐RADS 3–5 categories were combined into a single group for analysis. All of the above procedures were performed independently by two senior imaging diagnosticians with extensive postprocessing experience, and only those with consistent results were included in the study.

**Table 1:** Reference table for CAD‐RADS classification.

Degree of maximal coronary stenosis	CAD‐RADS categories
0% (No plaque or stenosis)	0
1%–24% (Minimal stenosis or plaque with no stenosis)	1
25%–49% (Mild stenosis)	2
50%–69%	3
70%–99%	4A
Left main >50% or three‐vessel obstructive (≥70%) disease	4B
100% (total occlusion)	5

Abbreviation: CAD‐RADS, coronary artery disease‐reporting and data system.

### 2.4 Statistical analysis of data

SPSS 26.0 statistical software was used for data analysis. The Kolmogorov–Smirnov method was used to test the normality of measurement data. Continuous variables conforming to the normal distribution are expressed as mean ± SD. The *t* test was used for comparison between the two groups. Counting data are expressed as %, and the chi‐squared test was used to compare the difference between the two groups. Univariate and multivariate logistic regression analyses were used to investigate the correlation between functional coronary artery ischemia and various variables. The predictive value of factors was evaluated through receiver operating characteristic (ROC) curves and area under the curve (AUC) analysis. The difference was statistically significant (*p* < .05).

## 3 RESULTS

### 3.1 Clinical data

There was no statistical difference in males, hypertension, current smoking, total cholesterol, or low‐density lipoprotein between the NAFLD group and the non‐NAFLD group (*p* > .05). Table 2 displays statistically significant differences in age, body mass index (BMI), hyperglycemia, hyperlipidemia, uric acid, triglyceride, and high‐density lipoprotein (*p* < .05).

**Table 2:** Comparison of clinical data between the non‐NAFLD group and the NAFLD group.

Variable	No NAFLD(*n* = 183)	NAFLD(*n* = 209)	*p* Value
Male	94 (51.4)	125 (59.8)	.093
Age	64.06 ± 11.13	60.88 ± 10.67	.004
BMI	24.19 ± 3.76	26.22 ± 3.14	.000
Hypertension	106 (57.9)	139 (66.5)	.080
Hyperglycemia	34 (18.6)	83 (39.7)	.000
Hyperlipidemia	92 (50.3)	147 (70.3)	.000
Current smoking	43 (23.5)	53 (25.4)	.669
UA	306.04 ± 82.25	340.92 ± 89.65	.000
TC	4.48 ± 1.06	4.48 ± 1.07	.997
TG	1.49 ± 0.10	2.10 ± 1.44	.000
HDLC	1.23 ± 0.28	1.11 ± 0.23	.000
LDLC	2.33 ± 0.79	2.37 ± 0.80	.651
CAD‐RADS classification			.019
1	38 (20.8)	28 (13.4)	
2	79 (43.2)	78 (37.3)	
3–5	66 (36.1)	103 (49.3)	
CACS classification			.030
1	32 (17.5)	25 (12.0)	
2	90 (49.2)	88 (42.1)	
3	61 (33.3)	96 (45.9)	
Number of diseased coronary arteries			.040
1	77 (42.1)	63 (30.1)	
2	53 (29.0)	67 (32.1)	
3	53 (29.0)	79 (37.8)	
CT‐FFR ≤ 0.80	71 (38.8)	110 (52.6)	.006

Abbreviations: BMI, body mass, index; CACS, coronary artery calcification score; CAD‐RADS, coronary artery disease‐reporting and data system; CT‐FFR, CT fractional flow reserve; HDLC, high‐density lipoprotein cholesterol; LDLC, low‐density lipoprotein cholesterol; NAFLD, nonalcoholic fatty liver disease; TC, total cholesterol; TG, triglyceride; UA, uric acid.

### 3.2 Image features

CAD‐RADS classification, CACS classification, number of diseased coronary arteries, and the constituent ratio of CT‐FFR ≤ 0.80 were significantly different between the two groups (*p* < .05) (Table 2).

### 3.3 Predictive factor of functional coronary artery ischemia in patients with NAFLD

**Figure 2:**
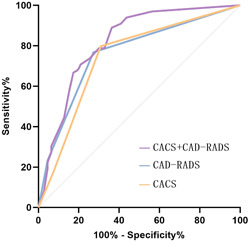
Receiver operating characteristic curve of each predictive factor. CACS, coronary artery calcification score; CAD‐RADS, coronary artery disease‐reporting and data system.

Univariate logistic regression analysis revealed a correlation between the number of diseased coronary arteries, CAD‐RADS classification, CACS classification, and patients with functional coronary artery ischemia in combination with NAFLD (*p* < .001). Further multivariate logistic regression analysis revealed CAD‐RADS classification and CACS classification as independent predictive factors for patients with functional coronary artery ischemia combined with NAFLD, and the incidence of functional coronary ischemia was positively correlated with CAD‐RADS classification (adjusted odds ratio [OR] = 3.383) and CACS classification (adjusted OR = 2.264) (Table 3). Based on the inclusion criteria of the ROC curve, the AUC of predicting coronary artery ischemia in patients combined with NAFLD was 0.762 for CAD‐RADS classification, 0.742 for CACS classification, and 0.819 for the combination of two indexes, with 95% confidence interval (CI) of 0.696–0.828, 0.673–0.811, and 0.761–0.876, respectively (all *p* = .000) (Figure 2).

**Table 3:** Univariate and multivariate regression analysis of functional coronary artery ischemia in patients with NAFLD.

Variable	Univariate analysis		Multivariate analysis	
	OR（95% CI）	*p* Value	OR（95%CI）	*p* Value
Male	0.983 (0.565 ~ 1.711)	.953		
Age	1.008 (0.982 ~ 1.034)	.557		
BMI	0.966 (0.886 ~ 1.054)	.966		
Hypertension	1.518 (0.852 ~ 2.705)	.156		
Hyperglycemia	1.471 (0.880 ~ 2.460)	.141		
Hyperlipidemia	1.274 (0.703 ~ 2.308)	.425		
Current smoking	1.884 (0.990 ~ 3.584)	.054		
UA	0.998 (0.995 ~ 1.001)	.276		
TC	0.926 (0.718 ~ 1.193)	.551		
TG	0.984 (0.814 ~ 1.189)	.864		
HDLC	1.069 (0.321 ~ 3.558)	.913		
LDLC	0.858 (0.611 ~ 1.206)	.379		
Number of diseased coronary arteries	2.452 (1.704 ~ 3.528)	.000	1.173 (0.734 ~ 1.874)	.505
CAD‐RADS classification	5.185 (3.118 ~ 8.623)	.000	3.383 (1.912 ~ 5.986)	.000
CACS classification	4.323 (2.633 ~ 7.100)	.000	2.264 (1.252 ~ 4.095)	.007

Abbreviations: BMI, body mass, index; CACS, coronary artery calcification score; CAD‐RADS, coronary artery disease‐reporting and data system; CI, confidence interval; NAFLD, nonalcoholic fatty liver disease; OR, odds ratio.

## 4 DISCUSSION

NAFLD is a disorder of fat metabolism. The fatty liver synthesizes and releases unsaturated fatty acids, lipid peroxide end products, and other atherogenic factors into the blood, contributing not only to the progression of fatty liver disease, but also to the development of atherosclerosis and coronary heart disease.^17^ NAFLD is associated with the metabolic syndrome, which includes obesity, insulin resistance, diabetes, and dyslipidemia.^18^ Studies^19^ indicate that hyperglycemia, hyperlipidemia, obesity, high uric acid, and other indexes of metabolic abnormalities are risk factors for NAFLD and that high‐density lipoprotein is a protective factor. The analysis of this investigation included common relevant factors. The results showed patients with NAFLD had a higher BMI, constituent ratio of hyperglycemia and hyperlipidemia, uric acid, and triglycerides compared to patients without NAFLD, and their high‐density lipoprotein levels were lower than those of patients without NAFLD, which is consistent with the results of previous studies. Furthermore, the results of this investigation revealed that the age of patients with NAFLD was lower than that of patients without NAFLD. This may be due to the fact that as age increases, NAFLD gradually progresses to end‐stage diseases such as cirrhosis and liver cancer or the death of patients with NAFLD due to the combination of other metabolic disorders, leading to a decrease in the number of patients with NAFLD, especially when combined with hepatitis B, which worsens the disease progression.

CAD‐RADS is a standardized reporting system for patient‐specific CCTA results. Realizing the standardization of reporting is beneficial to the standardized communication between radiologists and clinicians, thereby facilitating further patient management decisions. CACS is a method proposed by Agaston to detect and analyze the burden of coronary artery calcified plaque. Ren et al.^19^, ^20^ found a statistical difference in CACS classification and CAD‐RADS classification between the NAFLD group and the non‐NAFLD group. Patients with NAFLD had a higher constituent ratio of CACS ≥ 100 and CAD‐RADS 3–5 categories compared to the non‐NAFLD group, which is consistent with the results of our study. This indicates that patients with NAFLD are prone to severe coronary artery lesions, and NAFLD may even promote the progression of cardiovascular diseases,^21^ as confirmed by the study of Targer et al. In addition, we analyzed the constituent ratio of patients with CT‐FFR ≤ 0.80 in both groups, and the results indicated that patients with NAFLD had a higher probability of coronary artery functional ischemia.

In conclusion, we discovered that patients with NAFLD are more likely to develop cardiovascular diseases than patients without NAFLD. Rapid and accurate prediction of whether patients with NAFLD have combined coronary artery functional ischemia is of great clinical significance, as it is more beneficial in preventing or delaying the development and deterioration of coronary diseases in patients with NAFLD. Therefore, we further analyzed the predictive ability of clinical and imaging features of patients with NAFLD for coronary artery functional ischemia using noninvasive CT‐FFR technology. The incidence of coronary CT‐FFR ≤ 0.80 was associated with the number of diseased coronary arteries, CAD‐RADS classification, and CACS classification, as determined by univariate regression analysis. Additional multivariate regression analysis showed CAD‐RADS classification (adjusted OR = 3.383) and CACS classification (adjusted OR = 2.264) as independent predictive factors of the incidence of coronary artery functional ischemia. CAD‐RADS is primarily categorized based on the degree of coronary artery stenosis and plaque, and the degree of coronary artery stenosis is frequently highly correlated with the value of coronary CT‐FFR, which may be the reason why CAD‐RADS classification can predict coronary artery functional ischemia. According to the results of Detrano et al.,^21^, ^22^ CACS can enhance the predictive value of coronary artery ischemic events. Our results also indicate a positive correlation between CACS classification and the incidence of coronary artery functional ischemia in patients with NAFLD, indicating that the coronary artery ischemia group has a higher CACS than the nonischemia group, which is consistent with other results of the study.^23^, ^24^ Finally, we calculated the AUC values of CACS classification, CAD‐RADS classification, and the combination of the two indexes for predicting functional coronary artery ischemia in patients with NAFLD and demonstrated that the predictive value of the combination of multiple imaging indexes was higher than that of using a single index.^25^

This research has several limitations. This was a single‐center retrospective investigation, and the selection of sample size was limited to some extent. We plan to collaborate with other medical institutions to execute large‐scale prospective studies to increase the reliability of study results. In addition, the occurrence of coronary artery ischemic events may be influenced by a variety of factors, such as plaque characteristics, pericoronal fat attenuation index, and so on. We will include additional data to investigate more predictive factors for functional coronary artery ischemia in patients with NAFLD.

## 5 CONCLUSION

In conclusion, there were differences between patients with NAFLD and those without NAFLD. Furthermore, CAD‐RADS classification has a stronger predictive ability for coronary artery ischemia in patients with NAFLD, providing a theoretical basis for the early prevention or subsequent treatment of functional coronary artery ischemia in patients with NAFLD.

## AUTHOR CONTRIBUTIONS

*Conception and design of the research*: Hong‐Wei Xu. *Acquisition of data*: Wen‐Jing Li. *Analysis and interpretation of the data*: Wen‐Jing Li. *Statistical analysis*: Wen‐Jing Li. *Writing of the manuscript*: Wen‐Jing Li. *Critical revision of the manuscript for intellectual content*: Hong‐Wei Xu. All authors read and approved the final draft.

## CONFLICT OF INTEREST STATEMENT

The authors declare no conflict of interest.

## Data Availability

The data sets used and/or analyzed during the current study are available from the corresponding author on reasonable request.
